# Effects of Acute Salinity Stress on the Histological and Bacterial Community Structure and Function in Intestine of *Stichopus monotuberculatus*

**DOI:** 10.3390/md22120576

**Published:** 2024-12-23

**Authors:** Lianghua Huang, Hui Wang, Chuanyan Pan, Xueming Yang, Guoqing Deng, Yaowen Meng, Yongxiang Yu, Xiuli Chen, Shengping Zhong

**Affiliations:** 1 Institute of Marine Drugs, Guangxi University of Chinese Medicine, Nanning 530200, China; huanglh@gxtcmu.edu.cn (L.H.); dengguoqing135@163.com (G.D.); mywfami@163.com (Y.M.); 2 Guangxi Key Laboratory of Marine Drugs, Guangxi University of Chinese Medicine, Nanning 530200, China; 3 College of Ecology, Hainan University, Haikou 570228, China; 4 Guangxi Academy of Fishery Sciences, Nanning 530021, China; 13878177769@163.com (H.W.); gxscpcy@163.com (C.P.); nnyxming@126.com (X.Y.); 5 Key Laboratory of Maricultural Organism Disease Control, Yellow Sea Fisheries Research Institute, Chinese Academic of Fishery Sciences, Qingdao 266071, China

**Keywords:** *Stichopus monotuberculatus*, salinity stress, histological characteristics, intestinal flora, dominant bacteria

## Abstract

This study focused on *Stichopus monotuberculatus* and conducted stress experiments at salinity levels of 20‰ and 40‰. Intestinal histological changes and the structural characteristics of the intestinal flora of *S. monotuberculatus* under salinity stress were analyzed. The results show that acute salinity stress inflicts varying degrees of damage to the intestinal tissues of *S. monotuberculatus*. Salinity stress enhances the species diversity of intestinal flora in *S. monotuberculatus*. Eight phyla of bacteria are detected in the intestine of *S. monotuberculatus*. Dominant phyla include Proteobacteria, Firmicutes, and Actinobacteria. Furthermore, functional prediction reveals that acute salinity stress can significantly modify the abundance of pathways associated with nutrient and energy metabolism mediated by the intestinal flora of *S. monotuberculatus*. These results indicate that acute salinity stress induces pathological damage to the intestinal tissues of *S. monotuberculatus*, compromising the microbial habitat and leading to alterations in the intestinal flora composition. Additionally, *S. monotuberculatus* can mitigate salinity stress by adjusting the composition of its intestinal flora and the corresponding functional pathways.

## 1. Introduction

*S. monotuberculatus* belongs to the phylum Echinodermata, the class Holothurian, the order Pelargonidae, the family Stichopodidae, and the genus Stichopus. It is mainly distributed in the South China Sea and is commonly called “yellow meat ginseng” [1]. Sea cucumbers have important ecological functions in tropical marine ecosystems [2]. Coral reef habitat is an excellent habitat for sea cucumbers, and the ecological restoration function of sea cucumbers can effectively maintain the health of coral reef ecosystems [3]. As a sedimentary feeding marine animal, *S. monotuberculatus* promotes the recycling of sediment organic matter and strengthens the transfer of system energy flow and logistics [2]. In addition, the metabolites of *S. monotuberculatus* increased the productivity of coral reef communities and the health of coral reef ecosystems [2,4]. *S. monotuberculatus* serves as a typical sea cucumber in the tropical coral reef waters of southern China [5]. The tropical sea of the South China Sea is the most suitable sea area for the cultivation of *S. monotuberculatus*, due to its unique island and reef resources and excellent water quality [6]. In recent years, the government has built more marine ranchings, which provides good conditions for the cultivation of *S. monotuberculatus* [6]. The artificial breeding and seedling breeding techniques of *S. monotuberculatus* were the subject of a successful breakthrough in 2010 [7].

Salinity, as an important environmental factor in the marine ecosystem, plays a crucial role in the survival, growth, reproduction and enzyme activity and metabolic level of marine organisms, such as *Callinectes ornatus*, *Litopenaeus vannamei*, *Ruditapes philippinarum*, *Corbicula fluminea*, *Saxidomus purpurata*, *Trapezium liratum*, and *Pinctada fucata* [8,9,10,11,12]. Extreme salinity fluctuation will damage the physiological factors of marine annimals, resulting in death and economic loss in severe cases [13,14]. The intestine serves as a vital organ for digestion and absorption in aquatic animals, hosting a diverse and substantial microbial community that plays an integral role in various physiological functions, including the host’s energy metabolism [15,16]. Salinity change can not only disrupt the physiological structure of marine organisms but also affect the composition of their intestinal flora [17]. Due to its crucial role in the development and physiology of the intestinal tract, as well as in the body’s development, growth, and environmental adaptation, the intestinal flora is usually considered an “extra organ” [18]. The intestinal flora is an indispensable factor in nutrient absorption, material circulation, and physiological immunity of marine animals [19,20]. The balance of microbial communities significantly affects on the healthy growth and environmental adaptability of the host [21,22]. The structure and composition of the intestinal flora can vary with the host’s environmental conditions and dietary composition [23]. In aquatic animals, the intestinal flora is closely associated with the surrounding aquatic environment, rendering it particularly susceptible to physicochemical factors, such as temperature, salinity, and pH [24,25]. Existing research on intestinal flora and its benefits mostly focuses on vertebrates, especially mammals.

In recent years, high-throughput microbial sequencing technology based on 16S rDNA has been extensively applied in the study of microflora structure and become a key means of exploring the composition, structure, and function of microflora [26]. More and more scholars are adopting high-throughput sequencing technology to investigate the intestinal flora in marine animals. Zarkasi discovered that the intestinal flora of *Salmo salar* was mainly composed of Proteobacteria and Firmicutes [27]. Wei found that the dominant bacteria in the same batch of *Larimichthys crocea* with different weights and genders were Proteobacteria, Bacteroidetes, firmicutes and soficutes [28]. Studies on shrimps revealed that Proteobacteria was the dominant phylum in their intestines [29,30]. Regarding marine invertebrates, there have been massive studies on the microbiota of sponges and corals [31]. However, reports on the intestinal flora of other marine invertebrates are rare [32].

Research on the intestinal flora of echinoderms is mainly related to the structure and abundance of intestinal flora. For example, the compositions of the dominant intestinal flora of *Strongylocentrotus intermedius* with different feeding sources are basically consistent [33]. However, there are significant differences in relative abundance and metabolic pathways [33]. The types and proportions of dominant intestinal flora in *Apostichopus japonicus* vary with environmental factors [34]. Temperature can impact the colonization of bacteria of the genus Vibrio in the intestine of *A. japonicus* [35]. However, there is no relevant report on the intestinal flora of *S. monotuberculatus*, especially on the response and adaptation mechanisms under various salinity conditions.

This study observed structural changes in the intestinal tissues of *S. monotuberculatus* under salinity stress. Moreover, it analyzed the structural characteristics of the intestinal flora of *S. monotuberculatus* under different salinity stresses using 16S rRNA high-throughput sequencing technology. A comparative analysis was conducted to assess the diversity and structural changes of the intestinal bacteria in *S. monotuberculatus* under both high-salinity and low-salinity stress conditions. The objective is to elucidate the relationship between fluctuations in salinity and the health of intestinal tissues, as well as the composition of the intestinal flora in *S. monotuberculatus*, identify the dominant microbial taxa present in the intestines of *S. monotuberculatus* under varying salinity levels, and investigate the effects of different salinity conditions on the metabolic functions of the intestinal flora of *S. monotuberculatus*. This study will provide a theoretical basis for the healthy cultivation and the disease prevention and control of *S. monotuberculatus*.

## 2. Results

### 2.1. Intestinal Tissue Alternation

**Figure 1:**
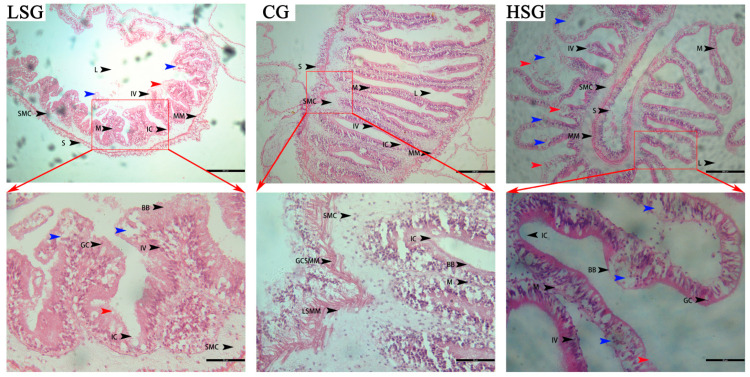
Transverse sections of intestinal tissues (foregut) HE staining of *S. monotuberculatus* under different salinity. LSG: low-salinity group; CG: control group; HSG: high-salinity group. IV: intestinal villi; L: lumen; IC: crypt; M: mucosa; SMC: submucosa; MM: muscularis layer; S: serosa layer; BB: brush border; GC: goblet cell; CSMM: circular smooth muscle; LSMM: longitudinal smooth muscle. The blue arrow indicates the presence of vacuolation in the intestine; the red arrow indicates death to epithelial cell of the intestine.

Salinity stress caused profound damage to the intestinal tissues (foregut) of *S. monotuberculatus*. Figure 1 shows the intestinal histological morphology of *S. monotuberculatus* after 96 h living in different salinity environments. The control group exhibited abundant, elongated villi that filled the intestinal lumen, along with deep crypts. In contrast, the salinity stress group showed reduced villus length and crypt depth, with compromised structural integrity at the microbial attachment sites compromised (Table 1). Notably, the brush border was absent in the high-salinity group, and the crypt architecture was severely disrupted. Furthermore, the thickness of both the mucosal and muscular layers decreased in the salinity stress groups, with varying degrees of degeneration and necrosis observed in epithelial cells (red arrow in Figure 1). In the high-salinity group, substantial epithelial cell death (red arrow in Figure 1) and vacuolation (blue arrow in Figure 1) were noted in certain regions. These results indicate that acute salinity stress has a deleterious impact on the intestinal tissues of *S. monotuberculatus*, leading to remarkable structural alterations and disruption of the microbial habitat within the gut.

### 2.2. Analysis of Operational Classification Units

**Figure 2:**
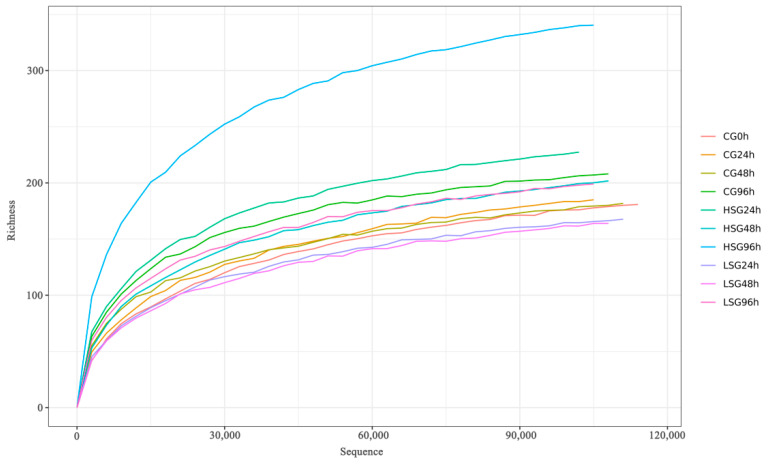
Sample dilution curve.

**Figure 3:**
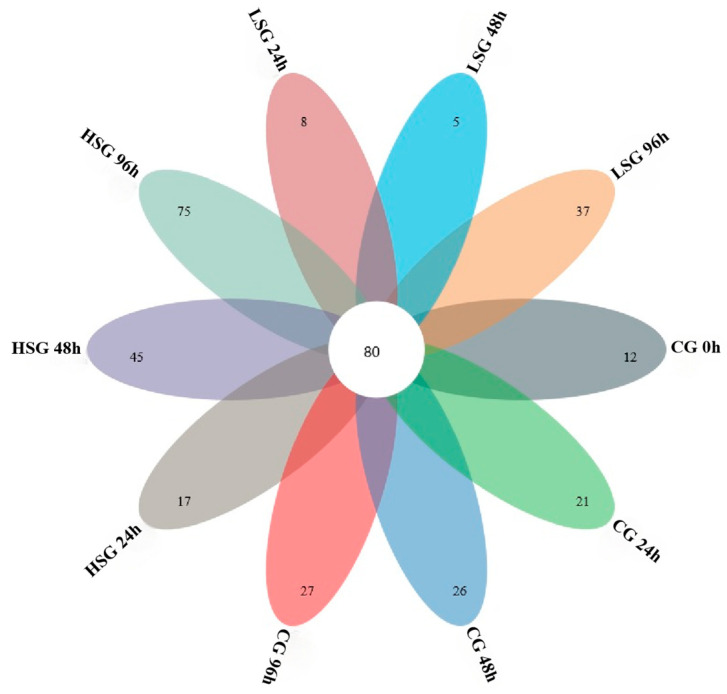
Venn diagrams of OTUs.

This study was based on 16S rRNA high-throughput sequencing technology to analyze the V3 and V4 regions of the intestinal flora of *S. monotuberculatus*. After quality control and the removal of low-quality and short sequences from ten groups of samples, 128,534 valid sequences on average were obtained for each sample, with an effective sequence of 120,628 and an effective sequence percentage of over 93.85%, indicating that the valid sequences derived from this sequencing can meet the requirements of the subsequent microbial diversity analysis (Figure 2). A total of 273 bacterial OTUs were identified through comparison with a bacterial classification database. Specifically, 130, 217, and 154 OTUs were identified in the low-salinity group (LSG), high-salinity group (HSG), and control group (CG), respectively. Among these, 80 OTUs were common to all groups, while the unique OTUs for each group were 50, 137, and 74, respectively (Figure 3). These findings imply that salinity stress modifies the OTU count of the intestinal flora in *S. monotuberculatus*.

### 2.3. Analysis of Intestinal Flora Diversity

#### 2.3.1. Alpha Diversity of Intestinal Flora

The indicators of Alpha diversity include Shannon, Simpson, Chao, and ACE. The coverage rates of the samples in the groups are all close to 1.0, with no significant difference (*p* > 0.05) (Table 2), representing a low probability of microbiota not sequenced in the samples, which can truly reflect the composition of intestinal microbiota in *S. monotuberculatus* under salinity stress. The sequencing depth basically covered all species in the samples. The possibility of new species is very small.

The Alpha diversity analysis results of the intestinal flora of *S. monotuberculatus* in different salinity environments are shown in Table 1. After the onset of salinity stress, the Shannon index shows a trend of first decreasing and then increasing. Meanwhile, the Simpson index exhibits a trend of first growing and then declining. The turning points are all at 48 h after salinity stress, indicating that the time of 48 h is a key node for the changes in the intestinal flora structure of *S. monotuberculatus* under salinity stress. After 96 h of salinity stress, the Shannon index and Simpson index of the low-salinity group are closer to the control group. After 24 h and 96 h of salinity stress, the Chao index and ACE index of the stress groups are higher than those of the control group, with the highest in the high-salinity group. This demonstrates that both the high-salinity group and the low-salinity group generated new microorganisms in the intestinal tract of *S. monotuberculatus* compared to the control group, resulting in a higher species richness of the intestinal flora.

#### 2.3.2. Beta Diversity of Intestinal Flora

**Figure 4:**
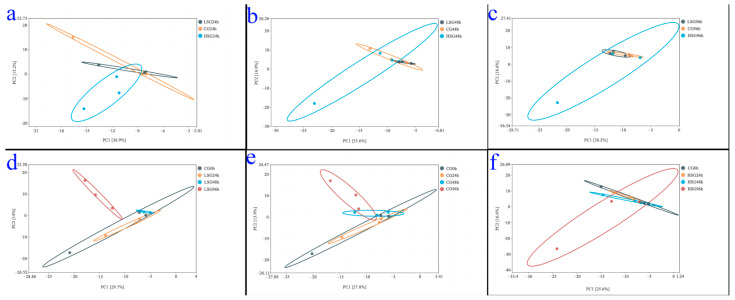
Beta diversity analysis index based on PCA analysis. Note: (**a**–**c**) respectively represent the PCA diagram of intestinal flora in *S. monotuberculatus* treated with different salinity for 24 h, 48 h and 96 h; (**d**–**f**) respectively represent the PCA diagram of intestinal flora in *S. monotuberculatus* treated with the same salinity and different treatment time, (**d**): low-salinity group; (**e**): control group; (**f**): high-salinity group.

The PCA analysis results of the intestinal flora of *S. monotuberculatus* in different salinity environments are shown in Figure 4. At the same stress time point, the low-salinity group and the control group basically clustered together, while the high-salinity group had large distances from the samples of the low-salinity group and the control group, implying significant differences in the intestinal flora structure of the high-salinity group’s *S. monotuberculatus* compared to the low-salinity group and the control group (Figure 4a–c). At the same salinity, the composition of intestinal flora alters with stress time. The intestinal flora structure of each group began to change after 48 h of stress. Among them, the control group had a relatively slight variation, while the salinity stress groups changed remarkably; after 96 h of stress, the trends of changes in intestinal flora structure were basically consistent between the low-salinity group and the control group, while the intestinal flora structure of the high-salinity group changed acutely (Figure 4d–f).

### 2.4. Analysis of Intestinal Flora Structure

#### 2.4.1. Structural Analysis of Intestinal Flora at the Phylum Level

**Figure 5:**
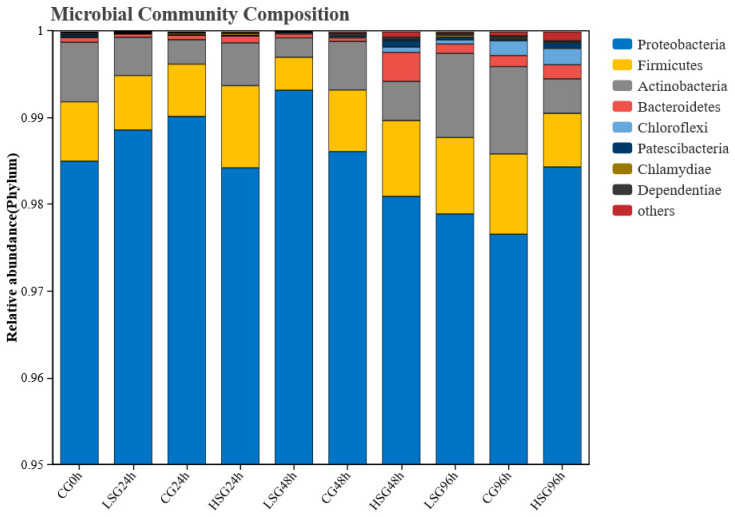
Relative abundance of bacterial community in the gut of *S. monotuberculatus* at the level of phylum.

At the phylum level in taxonomy, eight phyla are annotated for the intestinal flora of the samples in this study (Figure 5). Proteobacteria has the highest relative abundance, followed by Firmicutes and Actinobacteria. The samples at three stress time points are all dominated by Proteobacteria, the total abundance of which accounts for over 98% of the whole bacterial count. Evidently, with prolonged exposure to stress, the dominance of Proteobacteria, Firmicutes, and Actinobacteria present contrasting trends between the high-salinity and low-salinity groups.

#### 2.4.2. Structural Analysis of Intestinal Flora at the Genus Level

**Figure 6:**
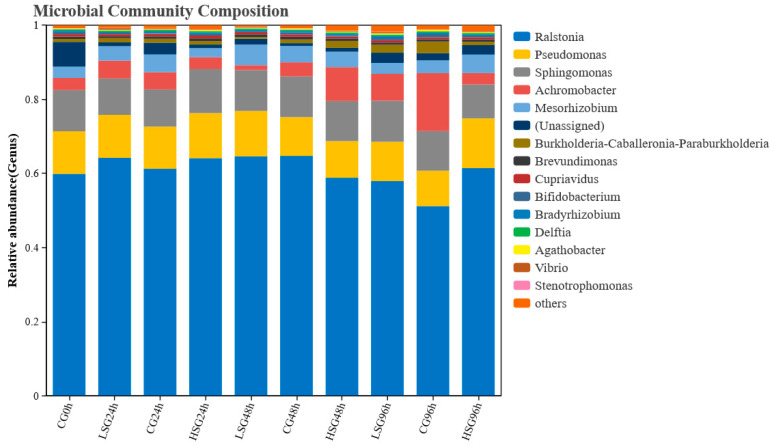
Relative abundance of bacterial community in the gut of *S. monotuberculatus* at the level of genus.

At the genus level, the floras with high relative abundance in the salinity stress groups and the control group of *S. monotuberculatus* mainly contain the genera of Ralstonia, Pseudomonas, Sphingomonas, Achromobacter, and Rhizobia (Figure 6), and the relative abundance of Ralstonia was about 60% in all groups. Throughout the entire experiment, the relative abundance of Ralstonia and Achromobacter changed the most, and both occurred after 48 h of treatment. Under salinity stress for 96 h, compared to the control group, the relative abundance of the Ralstonia genus elevated by 8.33% and 16.45%, that of the Achromobacterium genus dropped by 8.43% and 13.31%, and that of other bacteria was relatively stable.

### 2.5. Gene Function Prediction of Gut Bacterial Community

**Figure 7:**
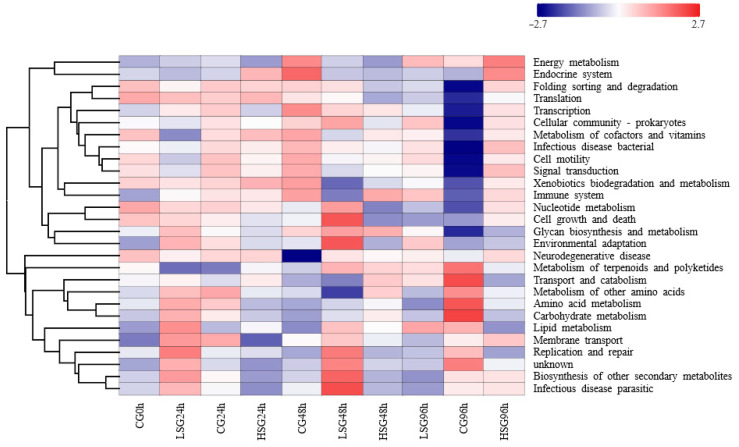
Heatmap showing the abundance distribution of potential functional pathways of gut bacterial communities in *S. monotuberculatus* under different salinity and different stress times. Note: The color from blue to red represents an increase in the abundance of the corresponding functional pathway; samples under different salinity stress were clustered according to functional pathways.

The predicted functional genes in the intestinal flora of *S. monotuberculatus* of the salinity-stress and control groups are annotated as seven major pathways and twentyy-eight minor pathways in the KEGG database, with an average abundance > 0.01%. The predicted functional profiles for all groups primarily involved the following aspects: among them, those related to metabolism account for the highest proportion (81.78%), and those associated with genetic information processing, cellular process, and environmental information processing also have relatively high abundance (>3.20%) in the bacterial community (Figure 7). After 48 h of salinity stress, marked differences in intestinal flora functions were observed between the low-salinity and high-salinity groups, primarily manifested in pathways related to membrane transport, environmental adaptation, lipid metabolism, energy metabolism, nucleic acid metabolism, and amino acid metabolism. Notably, the abundance of pathways for fatty acid synthesis, amino acid metabolism, and flagellar assembly was significantly higher in the low-salinity group compared to the high-salinity group (*p* < 0.05). After 96 h of salinity stress, the high-salinity group exhibited an apparently greater abundance of amino acid metabolism and fatty acid degradation pathways compared to the low-salinity group (*p* < 0.05). These findings suggest that the functional pathways mediated by intestinal flora differ significantly between high-salinity and low-salinity environments.

## 3. Discussion

Salinity is a critical factor influencing the survival, growth, development, and reproduction of marine animals. When salinity exceeds the adaptive range, it can trigger various stress responses in marine organisms, leading to tissue damage and mortality [36]. Echinoderms, being exclusively marine animals, are considered stenohaline due to their general inability to tolerate significant changes in seawater salinity [37,38]. Different species of echinoderms respond diversely to the challenge of the same salinity [39]. Specifically, the body wall, intestine, and respiratory trees of sea cucumbers respond rapidly to salinity fluctuations [40]. *S. monotuberculatus* is a tropical sea cucumber; however, how does its gut responds to acute salinity stress has not yet been reported.

The intestine functions as the primary digestive organ, and the anterior intestine demonstrates enhanced capabilities for digestion and absorption. Its morphology serves as a direct and prominent indicator of intestinal health [41]. The effectiveness of the digestive and absorptive processes in the intestines of marine animals is contingent upon the integrity of their tissue structures, which can be directly influenced by environmental factors [17,42]. The intestinal flora plays a critical role in maintaining intestinal microecology and facilitating the digestion and absorption of nutrients. A structurally intact intestine provides a stable foundation for the intestinal flora. The mucosal layer of the *S. monotuberculatus*’s intestine is responsible for secreting mucinous substances into the digestive tract, allowing for nutrient absorption via microvilli while storing and transporting nutrients to vascular cavities [43]. The thickness of this mucosal layer and the height of the villi are key determinants of the efficiency of nutrient digestion and absorption [44]. In this study, salinity stress resulted in a reduction of the mucosal layer thickness in the anterior intestine, shortening of the villi, and damage to the crypt structure, accompanied by varying degrees of degeneration and necrosis of epithelial cells, including vacuolation. More severe damage was observed in the high-salinity group. The disruption of the intestinal structural integrity compromises the habitat for gut microbes. Under salinity stress, bacterial habitat was destroyed, and the bacterial composition changed accordingly because the intestinal structure of *S. monotuberculatus* changed. Intestinal bacteria play an important role in the digestive system of sea cucumbers; they decompose large organic matter into small particles, and then produce small molecules that can be absorbed by the intestine to promote the absorption of nutrients [45]. Similar alterations in the intestinal tissue structure of *Apostichopus japonicas* have been documented in response to other environmental stressors [46,47]. Moreover, salinity stress has also been linked to diverse degrees of damage to the intestinal tissues of other marine organisms, such as *Sinonovacula constricta* [48], *Luciobarbus capito* [49], *lateolabrax maculatus* [50], and *Onchidium verruculatum* [51].

The intestine is the digestive organ of *S. monotuberculatus*, and intestinal flora is crucial for maintaining intestinal microecology and promoting the digestion and absorption of nutrients. The intestinal microbiota plays an important role in the host’s metabolism. Sudden variations in its structure and abundance can adversely impact organism health [52]. For instance, the colonization of microorganisms in the intestine of zebrafish promotes the absorption of fatty acids by epithelial cells [53]. Fishes with a complete microbiota exhibit increased lipid accumulation in the intestinal epithelium [54].

Changes in external environmental factors have a profound influence on the composition of intestinal flora in the body [55]. *S. monotuberculatus* is a sedimentary-feeding animal with a relatively simple digestive system. Its intestinal flora is mainly affected by the bacterial communities in the external environment. Moreover, the multi-layer structure of the intestinal tract provides a favorable living environment for the development and succession of bacterial communities [56,57]. Echinoderms are typical stenohaline marine animals and lack of salinity-regulating organs; *S. monotuberculatus* is extremely sensitive to salinity alternation in its living environment. Fluctuations in salinity can quickly cause responsive changes in its intestinal flora. In this study, salinity stress significantly impacted the intestinal flora structure of *S. monotuberculatus*. The key time node for the changes in the intestinal flora structure of *S. monotuberculatus* is 48 h of salinity stress. Meanwhile, salinity stress elevates the species diversity of intestinal flora in *S. monotuberculatus*, which may be due to differences in microbial communities in various salinity environments. In addition, with the prolongation of stress time, the changes in the composition and abundance of intestinal flora in the high-salinity group of *S. monotuberculatus* are more severe than those in the low-salinity group, which agrees with our previous research results that *S. monotuberculatus* has a stronger tolerance to low-salinity stress [58]. Gao detected 37 phyla of bacteria in the intestine of *A*. *japonicus* [59], while only a few bacteria were found in the intestine of sea urchins, and only abundant Proteobacteria existed [60,61]. In this study, a total of eight phyla of bacteria were annotated in the intestinal tract of *S. monotuberculatus*. The decrease in biodiversity may be attributed to the use of aerated tap water and seawater crystals as experimental water, resulting in fewer microbial species in the living environment of *S. monotuberculatus*.

Additionally, salinity stress profoundly affects the relative abundance of intestinal flora in *S. monotuberculatus*. The characteristics of the intestinal flora show that the phylum Proteobacteria has an absolute predominance. Proteobacteria is also the dominant phylum in the foreintestinal tract and intestinal tract of *A*. *japonicus*, with a ratio of up to 61% [32]. This phylum is also predominant in the intestinal microbiota of *S. monotuberculatus* exposed to sulfanilamide stress and in *Holothuria Scabra* fed different diets [62]. Clearly, Proteobacteria represent a common microbial constituent across various *S. monotuberculatus* species, indicating shared microbial communities within their intestines. Similar findings were reported by Xu and Weigel, demonstrating that Proteobacteria consistently colonize the intestines of *S. monotuberculatus* regardless of the aquaculture environment [63,64]. Research has shown that due to its strong environmental adaptability, Proteobacteria is the most abundant phylum in marine and aquatic environments, accounting for 79% of the deep-sea bacterial biomass, and widely exists in various aquatic organisms [65]. The advantages of Proteobacteria are consistent with previous studies on the intestinal bacterial composition of other marine invertebrates [66]. Proteobacteria are notable for their extensive species and genetic diversity, playing a critical role in the host’s nutritional metabolism [67]. They comprise the largest bacterial community in the culture system of *S. monotuberculatus*. Their abundance serves as an indicator of the host’s health, with an increase heightening the risk of disease in the host [68].

Firmicutes is a sub-dominant phylum in the intestinal tract of *S. monotuberculatus*, which is in accordance with the results of sea cucumber and the intestinal flora of sea cucumber [32,59]. The hallmark of Firmicutes is their strong adaptability to environmental conditions, enabling them to form resilient spores under stress. As environmental stressors intensify (e.g., low temperatures, high-salinity, and hypoxia), the relative abundance of Firmicutes tends to increase [69]. In this study, the elevated abundance of Firmicutes in *S. monotuberculatus* under salinity stress corroborates this conclusion. The increase in the relative abundance of Firmicutes is related to the accumulation of fat and the production of short-chain fatty acids (SCFA) [70]. Firmicutes promote the synthesis of SCFA and the decomposition of starch and dietary fiber. The above biological processes contribute to energy metabolism in hosts and enhance the immunity and adaptability to harsh environments [70].

In addition, this study also found that acute salinity stress caused changes in the relative abundance of intestinal flora, such as Ralstonia and Achromobacter in the intestinal tract of *S. monotuberculatus*. Ralstonia could lead to infection and death of many potentially harmful microorganisms and cause diseases such as ulcers in aquatic animals [71]. In this study, the relative abundance of Ralstonia in all groups showed an absolute advantage, indicating that the intestinal flora structure of *S. monotuberculatus* was relatively simple at this time, and the body was susceptible to changes in environmental factors, resulting in disease and even death of the organism [72]. Achromobacter has a strong corrupting effect on aquatic organisms, which can cause cystic fibrosis and damage intestinal health [73]. In this study, the relative abundance of Achromobacter changed dramatically under salinity stress, and its abundance increased with the extension of stress time, which was basically consistent with the results of intestinal tissue changes.

Changes in the intestinal flora of marine animals substantially impact the functional pathways they mediate [74]. Zhao et al. reported that fluctuations in aquatic environments rapidly induced responsive changes in the intestinal flora of sea cucumbers, which played a protective role for the host in adverse conditions [75]. In such a context, sea cucumbers prioritize withstanding the damage caused by extreme environments. They initiate as many functions as possible to fulfill this task, resulting in an upsurge in the abundance of bacteria (e.g., Proteobacteria) associated with environmental adaptation. In this study, salinity stress impacted the composition and diversity of the intestinal flora in *S. monotuberculatus*, leading to alterations in the metabolic functions mediated by intestinal flora. The PICRUSt analysis indicated that under both high-salinity and low-salinity stress conditions, the metabolic pathways mediated by the intestinal flora are similar, primarily involving fatty acid synthesis and amino acid metabolism. However, the abundance of these pathways varies between the two conditions. This is consistent with the metabolic pathways mediated by intestinal flora in *S. monotuberculatus* under extreme environmental conditions [46,76]. Furthermore, within both high-salinity and low-salinity environments, varying exposure times result in similar metabolic pathways, albeit with differing abundances. These findings suggest that *S. monotuberculatus* mobilizes additional energy metabolic pathways in response to salinity stress, utilizing greater resources to cope with the stress and mitigate potential adverse effects on the organism. The results of this study will help to improve the diversity and dynamic balance of intestinal flora in S. monotuberculatus, reduce the damage caused by salinity changes, and ensure its ecological role in tropical marine ecosystems. Although this study demonstrates the crucial role of intestinal flora in the response of *S. monotuberculatus* to extreme environments, further research is necessary to elucidate the underlying mechanisms at play.

## 4. Materials and Methods

### 4.1. Materials

The 1-year-old *S. monotuberculatus* individuals bred at the Zhanjiang Breeding Base of the Institute of Marine Medicine, Guangxi University of Traditional Chinese Medicine, were chosen for the experiment. After being transported back to the laboratory, the *S. monotuberculatus* individuals were raised in artificial seawater for two weeks. Among them, 200 healthy individuals with similar body sizes (weight: 3.23 ± 0.60 g, and body length: 32.26 ± 2.61 mm) were selected and transferred to the laboratory. Before the salinity stress experiment, they were domesticated in an intelligent recirculating aquaculture system for one week to reduce the interference of other factors except salinity in the stress experiments. *S. monotuberculatus* were fed a commercially formulated diet (Qingdao Hiford Ecological Technology Co., LTD, Qingdao, China) at 3% of body weight. During the domestication, the environmental parameters were maintained at 25 ± 1 °C, pH 8.0 ± 0.3, and DO > 5.0 mg/L by an intelligent recirculating aquaculture system.

### 4.2. Experiment Methods

#### 4.2.1. Experiment Design

The experiment set three groups: a low-salinity group (salinity: 20‰), a control group (salinity: 30‰), and a high-salinity group (salinity: 40‰). The water used in the experiment was artificial seawater, prepared by adding seawater crystals into fully aerated tap water to adjust to the target salinity. Each group had three replicates. Twenty individuals of similar size and good vitality [77] were randomly selected from each group and placed in a glass jar of approximately 0.064 m^3^ for closed inflatable breeding for 96 h. During the experiment, the commercially formulated diet and environmental parameters were the same as those of domestication.

#### 4.2.2. Sample Collection and Preparation

After starting the experiment, one *S. monotuberculatus* was randomly selected from each experimental cylinder at 0 h, 24 h, 48 h, and 96 h, respectively. The foregut of the *S. monotuberculatus* was quickly collected on the ice bag using a sterile dissector and placed in a 2 mL centrifuge tube. The samples were quickly frozen with liquid nitrogen and stored at −80 °C. For each group, three *S. monotuberculatus* individuals were selected to obtain the foregut, which were then fixed in 4% formaldehyde for 24 h prior to histological analysis.

#### 4.2.3. Histological Analysis of Intestinal Tissues

Following fixation, the foregut underwent a series of procedures, including dehydration through graded alcohol, clearing with xylene, paraffin embedding, continuous sectioning, Hematoxylin and eosin (HE) staining, and mounting. Observations and image capture were conducted using a Nikon Eclipse E100 microscope (Nikon Instruments Inc., Melville, NY, USA). Three sections were randomly selected from each group. Then, the intestinal villus length, crypt depth, mucosal layer thickness, and muscle layer thickness were randomly measured at 5 sites on each section using CaseViewer software (https://www.3dhistech.com/solutions/caseviewer/) (accessed on 18 December 2023) [78,79,80].

#### 4.2.4. Total DNA Extraction and Sequencing of Bacteria

The total DNA of the intestinal flora was extracted by the E.Z.N.A.^®^ Soil DNA Kit (Omega Bio-Tek, Inc., Norcross, Georgia). The DNA quality was detected using 10 g/L agarose gel electrophoresis, and the concentration and purity of the DNA were determined with Thermo NanoDrop One (ThThermo Fisher Scientific, Waltham, MA, USA). The process of gene amplification and sequencing was as follows: Based on the characteristics of the 16S rRNA gene sequence and the sequencing requirements of the Illumina MiSeq platform, PCR amplification was performed using universal primers 515F (5′-GTGCCAGCMGCCGCGGTAA-3′) and 806R (5′-GGACTACHVGGGTWTCTAAT-3′) (the primer fragment size: 470 bp; with barcode). The PCR reaction system (50 μL) was 2× Premix Taq 25 μL. Positive and negative primers (10 mmol/L) were 1 μL each. The sample DNA (20 ng/μL) was 50 ng, supplemented with nuclease-free water to 50 μL. PCR reaction conditions: 94 °C for 5 min; 94 °C for 30 s; 52 °C for 30 s; 72 °C for 30 s and 30 cycles; 72 °C for 10 min; stored at 4 °C for later utilization. The E.Z.N.A.^®^ GelExtraction Kit was adopted to recover PCR mixed products, and the T.E buffer was used for the elution and recovery of target DNA fragments. The library establishment was according to the standard process of the NEBNext^®^ ULTRATM DNA Library Prep Kit for Illumina^®^ (San Diego, CA, USA). After establishment, computer sequencing was carried out. PE250 sequencing was performed on the constructed amplicon library using the Illumina Nova 6000 platform.

#### 4.2.5. Data Processing Methods

OTU annotation and species annotation.

The Uparse algorithm (version 7.1; http://drive5.com/uparse/) (accessed on 18 December 2023) was employed to cluster the valid data of all samples. Based on the 97% similarity level, operational taxonomic unit (OTU) clustering was performed on the sequences. The RDP classifier Bayesian algorithm was used for the bioinformatics statistical analysis of OTUs under the 97% similarity level. Using QIIME software, the silva databases (Release128; http://www.arb-silva.de) (accessed on 18 December 2023) were compared and annotated at different classification levels to obtain the information on species classification corresponding to each OTU.

Alpha diversity analysis.

Qiime software (Version 1.9.1) was employed in Alpha diversity analysis to calculate the indicators of Shannon, Simpson, Chaol, ACE, and Goods-coverage [81]. R software (Version 2.15.3) was used to plot dilution curves and analyze inter-group differences in Alpha diversity indicators. Parametric and non-parametric tests were carried out simultaneously.

Beta diversity analysis.

Beta diversity calculates the distances between samples (groups) by analyzing the evolutionary relationships between sample sequences and abundance information, thereby reflecting the existence of significant differences in microbial communities between samples (groups). Principal Component Analysis (PCA) is a variance decomposition method using Euclidean distances to reduce the dimensions of multidimensional data and extract the most important elements and structures [82,83]. For Beta diversity analysis, this study used R software (Version 2.15.3) to draw PCA maps and Qiime software (Version 1.9.1) to calculate Unifrac distances. PCA analysis was conducted using the Ade4 package and gplot2 package of R software. Beta diversity index inter-group differences were analyzed with R software, and parametric and non-parametric tests were performed.

#### 4.2.6. KEGG Functional Analysis

The OTU abundance table was standardized with PICRUSt, eliminating the interference of copy numbers of 16S marker genes in the species genome. Subsequently, the green gene ID corresponding to each OTU was aligned to the KEGG database to derive information on KO, Pathway, and EC. Moreover, the abundance of each functional category was calculated according to the OTU abundance, resulting in abundance tables of samples at various taxonomic levels. Finally, the functional composition and abundance of each treatment group at specific functional classification levels were determined using cluster analysis.

#### 4.2.7. Statistical Analysis

All data were expressed as mean ± standard error. SPSS software 19.0 was used to test the normality, homogeneity of variance, and one-way analysis of variance (ANOVA). Duncan’s test was adopted to compare the differences between the groups, and *p* < 0.05 represented that the difference was statistically significant.

## 5. Conclusions

Salinity stress alters the intestinal tissue morphology of *S. monotuberculatus*, along with changes in the species diversity, composition, and potential metabolic functions of its intestinal flora. Under salinity stress, the intestinal structure of *S. monotuberculatus* was markedly compromised, disrupting the microbial habitat and leading to shifts in the intestinal flora composition. In response to these challenges, *S. monotuberculatus* adapt by modulating the composition of its intestinal flora. Notably, Proteobacteria emerged as the dominant phylum in the anterior intestine of *S. monotuberculatus* under salinity stress. A total of 48 h of stress was a key time node for the structural alternation of the intestinal flora. With the prolongation of stress time, changes in the composition and abundance of the intestinal flora of *S. monotuberculatus* in the high-salinity group were more intense than those in the low-salinity group. Additionally, under conditions of salinity stress, the metabolic pathways mediated by the intestinal flora in *S. monotuberculatus* were predominantly associated with energy metabolism, specifically involving fatty acid synthesis and amino acid metabolism. This study is the first to use high-throughput sequencing to analyze the intestinal flora of *S. monotuberculatus*. Along with other studies on the microbiota of echinoderms, it can be a basis for recognizing the intestinal flora ecology of marine invertebrates. Moreover, the composition and abundance variation of the intestinal flora in *S. monotuberculatus* under salinity stress were investigated for the first time in this study. These findings will provide a theoretical foundation for the healthy breeding and disease prevention of *S. monotuberculatus*. The next step will explore the role of the intestinal flora of *S. monotuberculatus* in environmental adaptation and immune defense.

## Figures and Tables

**Table 1:** The foregut histological parameters of *S. monotuberculatus* in different groups.

Parameters	LSG	CG	HSG
Villi length (μm)	122.66 ± 39.66 ^b^	124.08 ± 23.38 ^a^	119.64 ± 44.05 ^c^
Crypt depth (μm)	24.01 ± 6.34 ^ab^	25.32 ± 3.87 ^a^	21.07 ± 5.32 ^c^
Muscular layer thickness (μm)	4.86 ± 1.08 ^ab^	5.16 ± 0.86 ^a^	4.22 ± 2.25 ^c^
Mucosal layer thickness (μm)	123.74 ± 28.56 ^b^	125.49 ± 36.06 ^a^	118.05 ± 34.67 ^c^

Note: Measurement data expressed as the average of three independent measurements ± SD. Letters (a, b, c, ab) indicate the significant difference. Marked with the same letter or no letter indicates no significant difference between the mean values of treatment groups (Duncan’s test, *p* > 0.05), while marked with different letters shows significant difference between the mean values of treatment groups (Duncan’s test, *p* ˂ 0.05).

**Table 2:** Intestinal flora diversity index of *S. Monotuberculatus* at different salinity stress times.

Group	Shannon_2	Simpson	Chao1	ACE	Goods_Coverage
CG0h	2.06 ± 0.31 ^abc^	0.40 ± 0.06 ^ab^	183.17 ± 27.85	216.82 ± 30.56	0.9997 ± 0.00005
LSG24 h	1.98 ± 0.069 ^bc^	0.44 ± 0.01 ^a^	182.23 ± 11.61	211.02 ± 9.66	0.9997 ± 0.00005
CG24h	2.10 ± 0.15 ^abc^	0.41 ± 0.04 ^ab^	175.67 ± 16.95	208.12 ± 19.03	0.9996 ± 0.00004
HSG24h	1.99 ± 0.03 ^bc^	0.44 ± 0.01 ^a^	196.23 ± 5.41	232.19 ± 4.10	0.9996 ± 0.00001
LSG48h	1.90 ± 0.06 ^c^	0.45 ± 0.01 ^a^	160.1 ± 5.45	199.43 ± 10.43	0.9996 ± 0.00004
CG48h	1.96 ± 0.12 ^bc^	0.45 ± 0.02 ^a^	170.1 ± 16.14	204.14 ± 15.58	0.9997 ± 0.00002
HSG48h	2.19 ± 0.13 ^abc^	0.39 ± 0.04 ^ab^	196.33 ± 17.47	233.23 ± 17.22	0.9996 ± 0.00002
LSG96h	2.39 ± 0.10 ^ab^	0.37 ± 0.03 ^ab^	208.27 ± 7.04	242.80 ± 8.05	0.9996 ± 0.00003
CG96h	2.47 ± 0.09 ^a^	0.32 ± 0.02 ^b^	204.37 ± 8.12	242.49 ± 6.97	0.9996 ± 0.00005
HSG96h	2.19 ± 0.09 ^abc^	0.41 ± 0.01 ^ab^	210.6 ± 23.87	245.61 ± 27.39	0.9997 ± 0.00005

Note: Measurement data expressed as the average of three independent measurements ± SD. Letters (a, b, c, ab, bc, abc) indicate the significant difference. Marked with the same letter or no letter indicates no significant difference between the mean values of treatment groups (Duncan’s test, *p* > 0.05), while marked with different letters shows significant difference between the mean values of treatment groups (Duncan’s test, *p* ˂ 0.05).

## Data Availability

Data are contained within the article.
